# Contraceptive use and pregnancy planning in Britain during the first year of the COVID-19 pandemic: findings from a large, quasi-representative survey (Natsal-COVID)

**DOI:** 10.1136/bmjsrh-2022-201763

**Published:** 2023-03-23

**Authors:** Andrew J Baxter, Rebecca S Geary, Emily Dema, Raquel Bosó Pérez, Julie Riddell, Malachi Willis, Anne Conolly, Laura L Oakley, Andrew J Copas, Jo Gibbs, Christopher Bonell, Pam Sonnenberg, Catherine H Mercer, Soazig Clifton, Nigel Field, Kirsten Mitchell

**Affiliations:** 1 MRC/CSO Social and Public Health Sciences Unit, School of Health and Wellbeing, University of Glasgow, Glasgow, UK; 2 Institute of Population Health, University of Liverpool, Liverpool, UK; 3 Institute for Global Health, University College London, London, UK; 4 NatCen Social Research, London, UK; 5 Centre for Fertility and Health, Norwegian Institute of Public Health, Oslo, Norway; 6 London School of Hygiene and Tropical Medicine, London, UK

**Keywords:** reproductive health, sexual health, COVID-19, family planning services

## Abstract

**Background:**

Contraceptive services were significantly disrupted during the COVID-19 pandemic in Britain. We investigated contraception-related health inequalities in the first year of the pandemic.

**Methods:**

Natsal-COVID Wave 2 surveyed 6658 adults aged 18–59 years between March and April 2021, using quotas and weighting to achieve quasi-representativeness. Our analysis included sexually active participants aged 18–44 years, described as female at birth. We analysed contraception use, contraceptive switching due to the pandemic, contraceptive service access, and pregnancy plannedness.

**Results:**

Of 1488 participants, 1619 were at risk of unplanned pregnancy, of whom 54.1% (51.0%–57.1%) reported routinely using effective contraception in the past year. Among all participants, 14.3% (12.5%–16.3%) reported switching or stopping contraception due to the pandemic. 3.2% (2.0%–5.1%) of those using effective methods pre-pandemic switched to less effective methods, while 3.8% (2.5%–5.9%) stopped. 29.3% (26.9%–31.8%) of at-risk participants reported seeking contraceptive services, of whom 16.4% (13.0%–20.4%) reported difficulty accessing services. Clinic closures and cancelled appointments were commonly reported pandemic-related reasons for difficulty accessing services. This unmet need was associated with younger age, diverse sexual identities and anxiety symptoms. Of 199 pregnancies, 6.6% (3.9%–11.1%) scored as ‘unplanned’; less planning was associated with younger age, lower social grade and unemployment.

**Conclusions:**

Just under a third of participants sought contraceptive services during the pandemic and most were successful, indicating resilience and adaptability of service delivery. However, one in six reported an unmet need due to the pandemic. COVID-induced inequalities in service access potentially exacerbated existing reproductive health inequalities. These should be addressed in the post-pandemic period and beyond.

**WHAT IS ALREADY KNOWN ON THIS TOPIC**

- The COVID-19 pandemic likely impacted reproductive outcomes in diverse ways; such impacts may have been unequally distributed.
- Previous studies reported adaptations to health service delivery and difficulties experienced in accessing reproductive health services, with switching and stopping of contraceptive methods and potentially greater risk of unplanned pregnancy.

**WHAT THIS STUDY ADDS**

- We examined differences in contraceptive use and pregnancy planning in a sample of women, trans and non-binary people able to become pregnant who were quasi-representative of the British general population.
- We found that key markers of inequality and vulnerability, related to age, ethnicity, social disadvantage and mental health, were associated with increased contraceptive method switching, unmet need of contraceptive services and less-planned pregnancies.

**HOW THIS STUDY MIGHT AFFECT RESEARCH, PRACTICE OR POLICY**

- Ongoing efforts to ease the health impacts of the pandemic should aim to improve equality of access to contraceptive services.

## Introduction

The COVID-19 pandemic prompted rapid adjustment to health services, including the suspension or reduction of face-to-face consultations, increased remote provision, and rearranged appointments due to staff unavailability.^1 2^ Adjustments to contraceptive services included recommendation of methods not requiring face-to-face consultations (eg, the progestogen-only pill), amended guidance on off-label extended use of some long-acting reversible contraceptives (LARCs), and streamlined remote repeat prescribing of the combined contraceptive pill.^3^

Although aiming for equitable access,^4 5^ rapid adaptions during the pandemic had the potential to exacerbate inequalities, particularly if these required digital access and literacy.^6 7^ Service users might also interpret adaptations as de-prioritising contraceptive services,^8^ and we know that some patients self-censored their needs or were anxious about COVID-19 risk if accessing services in person.^9^ Overall, people in the UK and globally struggled to access contraception during lockdowns,^1 10–14^ and prescribing data for the UK showed substantial drops in LARCs fitted in 2020 versus 2019.^15^ Several studies suggest that young people were disproportionately affected by service closures.^10 16^

However, the pandemic’s effect on contraception and service use remains poorly understood. Previous studies have indicated difficulties accessing contraception, alongside changing sexual risk behaviours; however, these often used small convenience samples in the early stages of the pandemic.^8 14 16 17^ The Natsal-COVID study, a large national survey of sexual and reproductive health (SRH), was set up to address gaps in representativeness of studies and a lack of detailed information about the ongoing effects of the pandemic. Wave 1 findings (conducted 4 months after the first UK national lockdown) suggested young women were most likely to switch contraceptive and face barriers to sexual health service access.^11^ We have also reported Wave 2 findings that one in ten female participants had stopped or switched contraceptive method in the year after the first lockdown.^18^ In this study, we investigate inequalities in reproductive health service access, contraceptive method switching due to the pandemic, and pregnancy ‘plannedness’ during the first year of the pandemic among women, and trans and non-binary people who can become pregnant.

## Methods

### Study design and participants

Natsal-COVID Wave 2 is a quasi-representative web panel survey of SRH in Britain. Following the initial Natsal-COVID-1 data collection in July-August 2021, Wave 2 survey data were collected between 27 March 2021 and 26 April 2021 to capture SRH behaviour and outcomes during the first year of the COVID-19 pandemic.^19 20^ Participants aged 18–59 years answered an online questionnaire administered by Ipsos (median length 13 min). In addition to demographic and behavioural factors, participants were asked about SRH and service use in the period before the first UK lockdown in March 2020 and in the past year. The questionnaire is available online.^21^

We used sampling target quotas set by gender, age, region and social grade and weighting based on these demographics plus ethnicity and sexual orientation to achieve a quasi-representative sample of the British general population.^19 20^

### Statistical analysis

The Wave 2 sample (n=6658) comprised 2098 recontacted Wave 1 participants and 4560 new participants aged 18–59 years. The latter included a boost of 500 people aged 18–29 years, ensuring an overall sample of 2000 participants in this age group, who are often at greater risk of adverse SRH outcomes.^19^

To examine the impacts of the COVID-19 pandemic on pregnancy planning, we analysed prevalence and plannedness of pregnancy among participants aged 18–44 years who were described as female at birth and reported any sexual contact with a man since the start of the first UK lockdown (23 March 2020). Twenty-five participants reported a pregnancy in the past year but did not report sex with a man. These may have predominantly been conceptions occurring before the first lockdown and were not included in the sample. We analysed contraceptive method use and service access among a subsample of those at risk of unplanned pregnancy that we defined by excluding those currently pregnant, currently trying to conceive or not able to get pregnant.

To measure inequalities, we used educational attainment by highest academic qualification reported, and social grade based on occupation. Participants were classified as having symptoms of depression or anxiety if scoring >3 on the two-item Patient Health Questionnaire-2 (PHQ-2) or Generalised Anxiety Disorder-2 (GAD-2) screening tools, respectively.^22 23^

We categorised contraceptive methods by their effectiveness in preventing pregnancy based on typical-use failure rates (online supplemental box 1).^24 25^ We analysed emergency contraceptive (EC) use separately, assuming that changes in motivation and access would have affected use of planned methods and EC differently. Participants using another method in addition to EC were classified by the effectiveness of the non-emergency method; participants who only used EC were classified as using ‘no method’ for the purposes of prophylactic method-use comparisons. Unmet need for contraceptive services was defined as reporting trying but being unable to use contraceptive services. ‘Plannedness’ of pregnancies in the past year was estimated using the London Measure of Unplanned Pregnancy (LMUP, 2020 version),^26 27^ comprising questions on contraceptive use, timing of motherhood, intention to become pregnant, desire for a baby, discussion with partner and preconception preparations.^28^ Each item is scored 0–2 (summing to a total, range 0–12), with each point representing an increase in pregnancy ‘plannedness’. Scores of 0–3 were categorised as ‘unplanned’, 4–9 as ‘ambivalent’ and above 9 as ‘planned’. Full definitions for outcome variables and the denominators used in each analysis are given in online supplemental table S2.

10.1136/bmjsrh-2022-201763.supp1 Supplementary data

We used complex survey analysis functions in Stata (version 17.0). Figures were constructed in R (version 4.2.1).^29^ Weighted estimates are presented with weighted and unweighted denominators and unweighted numerators. We used the survey-equivalent chi-square statistic to determine whether there was statistically significant variation by sociodemographic and behavioural factors in the reported contraceptive method used since the start of the first lockdown and in the switching of contraceptive methods. We compared odds of using EC pre- and post-lockdown, using a conditional logistic regression model to account for intra-person clustering. We used logistic regression to calculate age-adjusted odds ratios (aORs) to investigate how use of, and unmet need for, contraceptive services varied by sociodemographic and behavioural factors. We used linear regression with robust standard errors to investigate differences in mean ‘plannedness’ of pregnancy scores and logistic regression to estimate differential odds of an ‘unplanned pregnancy’, adjusting for age. Proportions of missing demographic variables were relatively low, ranging from 0% to 1.3%; all comparisons were restricted to complete cases across relevant variables.

Natsal-COVID was approved by ethics committees at the University of Glasgow (20019174) and the London School of Hygiene and Tropical Medicine (22565). Participants provided informed consent electronically at the start of the survey.

### Patient and public involvement

Patients or the public were not directly involved in the design, conduct, reporting or dissemination plans of the Natsal-COVID Study due to the urgency of the research during the pandemic. However, members of the public were involved in the design of the Natsal-4 questionnaire, on which the Natsal-COVID questionnaire was based.

### Data availability

The data used in this study are available via the UK Data Service online catalogue.^30^

## Results

Of 6658 participants in Natsal-COVID Wave 2, 1488 were aged 18–44 years, described as female at birth and reported sexual contact with a man in the past year (online supplemental figure S1). Of these, most identified as ‘female’ (weighted proportion: 99.0%), two described themselves as ‘male’ and ten described themselves ‘in another way’. Most participants were White (86.7%), married or in a steady cohabiting relationship (70.6%) and identified as heterosexual (96.7%; online supplemental table S1).

**Figure 1:**
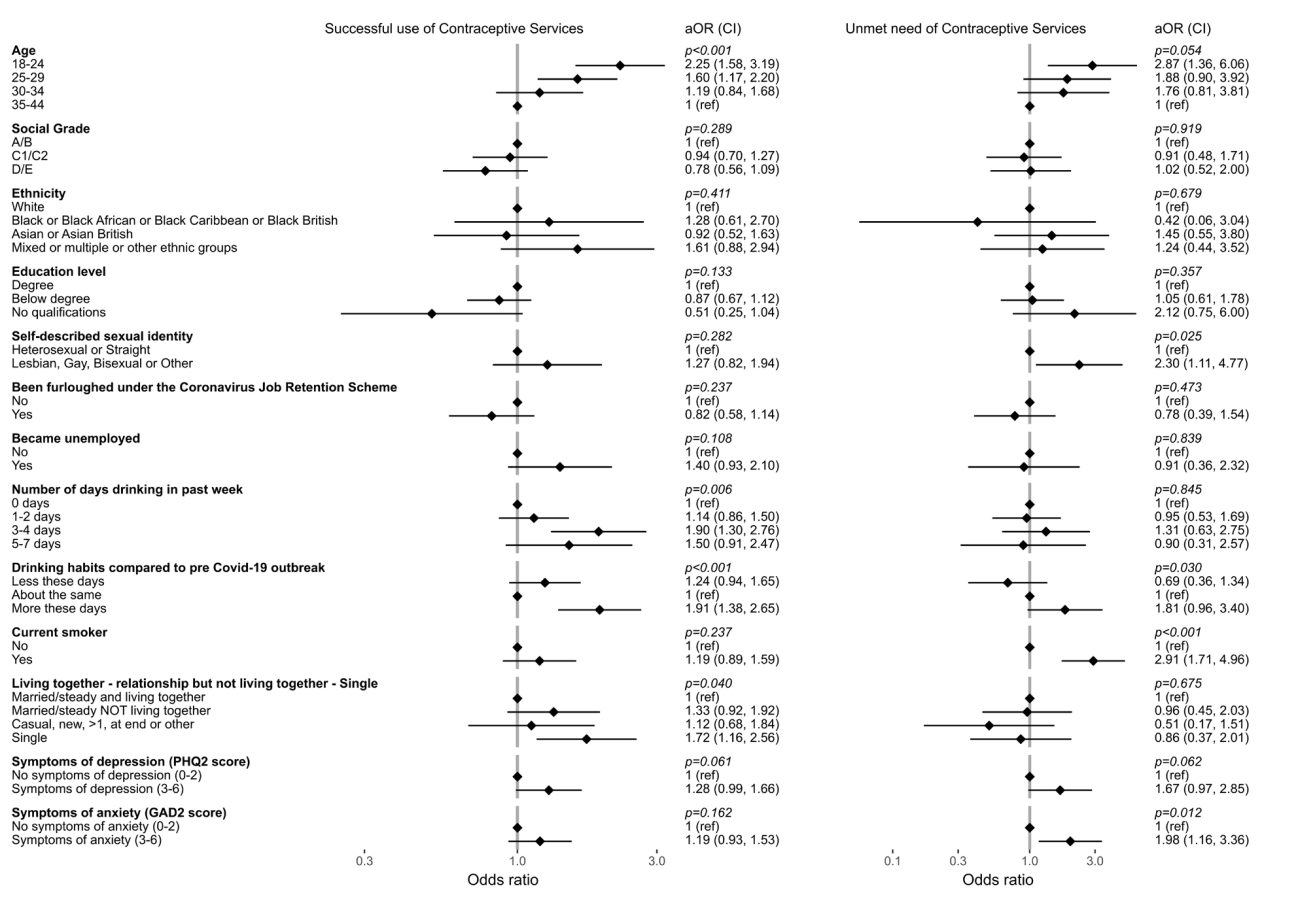
Contraception service access outcomes among all participants (n=1488) – factors associated with success and unmet need. All odds ratios are age-adjusted, except those for the age categories which are crude. Analyses are conducted across 441 participants (29.6%) who attempted to access a contraceptive service at least once since the start of the first lockdown. Social grade codes: A/B - Higher/intermediate managerial, administrative and professional; C1/C2 - Supervisory, clerical and junior managerial, administrative and professional, skilled manual workers; D/E - Semi-skilled and unskilled manual, casual, lowest grade and unemployed. aOR, adjusted odds ratio; CI, confidence interval.

Of 1415 participants who provided information about contraceptive use, 82.4% (unweighted n=1169; online supplemental figure S1) were deemed to be at risk of unplanned pregnancy (after excluding 150 who were currently pregnant or currently trying to conceive and 96 who were not able to get pregnant). Just over half of the participants at risk reported using a more effective contraceptive method as their usual or only contraceptive method in the past year (54.1%; table 1). This was lowest among participants aged 18–24 years (45.7% (38.7%–53.0%)), and considerably lower for participants from Black (27.6% (15.1%–45.0%)), Asian or Asian British ethnic backgrounds (25.9% (15.8%–39.4%)) or from a mixed or multiple or other ethnic background (27.5% (15.2%–44.7%)) than participants from White ethnic backgrounds (58.1% (54.7%–61.3%)). Those with at least one marker of lower socioeconomic status (working in less skilled occupations, receiving state benefit or unemployed at the time of survey) were less likely to report using a more effective method as their only/usual contraceptive method (D/E social grade: 42.9% (36.7%–49.2%) vs 60.9% (56.6%–65.0%) of C1/C2 grade). However, economic factors potentially related to the COVID-19 pandemic were not associated with effective contraception use (becoming unemployed: p=0.20 or furloughed: p=0.90; table 1). Among those at risk of pregnancy, reported EC use was higher in the year preceding the pandemic (reported by 3.4% (2.4%–4.9%)) than in the year from the start of the first lockdown (1.9% (1.1%–3.2%); conditional OR: 0.30 (0.12–0.75); data not shown).

**Table 1:** Contraception used in the year since the start of the first UK lockdown by participants aged 18–44 years who were sexually active and were not pregnant, not trying to get pregnant nor unable to get pregnant

Parameter	Usual contraception used during COVID-19 (% (95% CI))			Denominators (weighted, unweighted)
	No method used	Less effective method	More effective method	
Total	12.8 (10.9 to 15.0)	33.1 (30.3 to 36.1)	54.1 (51.0 to 57.1)	999,1169
Age (years)				
18–24	12.2 (8.2 to 17.8)	42.0 (35.1 to 49.3)	45.7 (38.7 to 53.0)	185, 230
25–29	14.9 (11.0 to 19.8)	28.7 (23.4 to 34.6)	56.5 (50.3 to 62.4)	255, 315
30–34	12.4 (8.6 to 17.6)	33.1 (27.1 to 39.8)	54.5 (47.7 to 61.1)	212, 252
35–44	11.8 (8.8 to 15.7)	31.7 (27.0 to 36.8)	56.5 (51.2 to 61.6)	346, 372
P value				p=0.10
Ethnicity				
White	11.9 (9.9 to 14.3)	30.0 (27.0 to 33.1)	58.1 (54.7 to 61.3)	863, 1040
Black or Black African or Black Caribbean or Black British	30.2 (17.0 to 47.6)	42.2 (26.8 to 59.3)	27.6 (15.1 to 45.0)	35, 27
Asian or Asian British	19.0 (10.5 to 32.0)	55.1 (41.5 to 68.0)	25.9 (15.8 to 39.4)	54, 49
Mixed or multiple or other ethnic groups	10.7 (3.9 to 26.2)	61.8 (44.7 to 76.4)	27.5 (15.2 to 44.7)	36, 47
P value				p<0.0001
Self-described sexual identity				
Heterosexual or Straight	13.0 (11.0 to 15.2)	33.3 (30.4 to 36.3)	53.8 (50.6 to 56.9)	952, 1035
Lesbian, Gay, Bisexual or Other	10.2 (3.5 to 26.6)	29.1 (16.0 to 47.1)	60.6 (42.9 to 76.0)	34, 123
P value				p=0.40
Social grade				
A/B - Higher/intermediate managerial, administrative and professional	11.7 (8.1 to 16.5)	38.1 (32.1 to 44.6)	50.2 (43.8 to 56.6)	233, 292
C1/C2 - Supervisory, clerical and junior managerial, administrative and professional, skilled manual workers	10.4 (8.1 to 13.3)	28.7 (25.0 to 32.8)	60.9 (56.6 to 65.0)	526, 582
D/E - Semi-skilled and unskilled manual, casual, lowest grade and unemployed	19.2 (14.7 to 24.7)	37.9 (32.0 to 44.3)	42.9 (36.7 to 49.2)	240, 295
P value				p<0.0001
Education level				
Degree	9.0 (6.8 to 11.8)	36.2 (32.2 to 40.5)	54.8 (50.4 to 59.0)	519, 609
Below degree	15.3 (12.3 to 19.0)	30.1 (26.0 to 34.6)	54.5 (49.9 to 59.1)	445, 520
No qualifications	37.4 (22.7 to 54.9)	25.3 (13.3 to 42.7)	37.4 (22.6 to 54.9)	35, 40
P value				p<0.0001
Living together – Relationship but not living together – Single				
Married/steady and living together	13.5 (11.2 to 16.3)	32.2 (28.8 to 35.8)	54.3 (50.5 to 58.0)	678, 781
Married/steady not living together	10.7 (6.4 to 17.3)	33.6 (25.9 to 42.2)	55.8 (47.1 to 64.1)	131, 164
Casual, new, >1, at end or other*	10.0 (5.0 to 19.0)	42.3 (31.8 to 53.5)	47.7 (36.8 to 58.7)	80, 92
Single	13.0 (7.8 to 20.8)	31.3 (23.2 to 40.7)	55.7 (46.2 to 64.8)	109, 131
P value				p=0.64
Been furloughed under the Coronavirus Job Retention Scheme				
No	13.0 (10.9 to 15.5)	33.3 (30.2 to 36.6)	53.7 (50.2 to 57.1)	822, 962
Yes	11.8 (7.8 to 17.7)	33.0 (26.3 to 40.5)	55.2 (47.6 to 62.5)	169, 200
P value				p=0.90
Became unemployed				
No	12.8 (10.8 to 15.2)	32.4 (29.4 to 35.6)	54.8 (51.5 to 58.0)	891, 1040
Yes	12.8 (7.5 to 20.9)	40.8 (31.6 to 50.8)	46.4 (36.8 to 56.3)	101, 122
P value				p=0.20
Days drinking in the last week (n)				
0	15.1 (11.9 to 18.9)	31.6 (27.3 to 36.3)	53.3 (48.4 to 58.1)	409, 474
1–2	10.6 (7.9 to 14.0)	32.4 (28.0 to 37.2)	57.0 (52.1 to 61.8)	396, 468
3–4	10.1 (6.0 to 16.7)	41.0 (32.8 to 49.7)	48.8 (40.3 to 57.5)	130, 149
5–7	16.2 (8.8 to 27.8)	32.3 (21.7 to 45.1)	51.5 (39.0 to 63.8)	62, 76
P value				p=0.18
Drinking habits compared with pre-COVID-19 outbreak				
Less these days	13.1 (9.7 to 17.5)	32.9 (27.8 to 38.5)	54.0 (48.3 to 59.6)	298, 360
About the same	13.0 (10.4 to 16.2)	32.2 (28.3 to 36.4)	54.8 (50.4 to 59.0)	510, 589
More these days	9.6 (6.0 to 14.9)	34.4 (27.7 to 41.8)	56.0 (48.6 to 63.2)	176, 205
P value				p=0.79
Current smoker				
No	11.2 (9.1 to 13.6)	33.3 (30.0 to 36.7)	55.6 (52.0 to 59.0)	774, 906
Yes	18.6 (14.0 to 24.3)	32.4 (26.5 to 38.8)	49.0 (42.4 to 55.6)	222, 261
P value				p=0.011
Symptoms of depression (PHQ-2 score)				
No symptoms of depression (0–2)	10.2 (8.1 to 12.8)	33.3 (29.7 to 37.0)	56.5 (52.6 to 60.3)	642, 745
Symptoms of depression (3–6)	17.9 (14.2 to 22.4)	33.3 (28.5 to 38.4)	48.8 (43.5 to 54.1)	346, 413
P value				p=0.0016
Symptoms of anxiety (GAD-2 score)				
No symptoms of anxiety (0–2)	10.4 (8.2 to 13.1)	33.2 (29.6 to 37.1)	56.4 (52.4 to 60.3)	607, 697
Symptoms of anxiety (3–6)	16.7 (13.3 to 20.8)	32.5 (28.0 to 37.3)	50.8 (45.8 to 55.8)	384, 465
P value				p=0.013

Some 246 respondents (17.4% of total) answered ‘Not applicable’ as they were already pregnant, planning to get pregnant or unable to get pregnant. These responses are excluded from the table. ‘More effective method’ includes intrauterine device, intrauterine system, implant, contraceptive pill, injection and transdermal patch. ‘Less effective method’ includes condoms, spermicides, rhythm method, withdrawal and ‘other’ methods. Participants who used no contraceptives or only emergency contraceptives are classed as ‘no method used’. P values were calculated from F values generated from Pearson χ^2^ statistics using the second-order correction of Rao and Scott.^45^

*In a ‘casual’ relationship, in a ‘new’ relationship, in more than one relationship, recently ended a relationship or ‘other’ relationship status.

CI, confidence interval; GAD-2, Generalised Anxiety Disorder-2; PHQ-2, Patient Health Questionnaire-2.

Overall, 12.8% (10.9%–15.0%) of participants at risk of an unplanned pregnancy reported no usual contraception methods (table 1). This was more likely in those who reported smoking (p=0.011), lower educational qualification (p<0.0001) and poor mental health (depression: p=0.0016; anxiety: p=0.013).

In total, 227 participants (14.3% (12.5%–16.3%) of participants included in this analysis) reported stopping or switching contraceptive method due to the pandemic. For all users of contraceptives before the start of the pandemic, stopping and switching directions are reported in online supplemental table S4. Among those using a more effective contraceptive at the start of the pandemic, 10.2% (7.9%–13.1%) reported switching to a similarly or more effective method, 3.2% (2.0%–5.1%) switched to a less effective method and 3.8% (2.5%–5.9%) stopped (table 2). Among users of effective methods, we found differences in stopping/switching by age, ethnicity and sociodemographic factors. Those aged 18–24 years were more likely than older participants to have switched method (23.8% (15.4%–34.8%) vs aged 25–44 years: 11.7% (9.0%–15.0%)). Compared with White participants, Black participants were more likely to have switched their usual method (29.6% (9.2%–63.7%) vs 11.8% (9.4%–14.8%)) and to have stopped using contraceptives (9.7% (1.2%–49.3%) vs 3.7% (2.3%–5.8%)). Reporting depression was associated with switching method (20.0% (14.6%–26.8%) vs 10.0% (7.2%–13.6%); table 2).

**Table 2:** Switching due to the pandemic from usual pre-COVID-19 contraception method among participants who were using ‘more effective’ contraceptives and were not pregnant, not trying to get pregnant nor unable to get pregnant

Parameter	Used more effective methods in the year before first lockdown and stopped or switched method (% (95% CI))				Denominators (weighted, unweighted)
	Did not switch or stop usual method	Switched to similarly or more effective method	Switched from more effective usual method to less effective method	Stopped using contraceptives	
Total	82.8 (79.3 to 85.8)	10.2 (7.9 to 13.1)	3.2 (2.0 to 5.1)	3.8 (2.5 to 5.9)	521, 631
Age (years)					
18–24	74.6 (63.5 to 83.3)	16.0 (9.3 to 26.2)	7.7 (3.4 to 16.5)	1.6 (0.3 to 9.1)	76, 102
25–29	80.9 (73.6 to 86.5)	9.2 (5.4 to 15.1)	3.9 (1.7 to 8.6)	6.1 (3.2 to 11.4)	146, 189
30–34	81.5 (73.0 to 87.7)	12.5 (7.5 to 20.2)	2.9 (1.0 to 8.4)	3.1 (1.1 to 8.6)	111, 133
35–44	88.3 (82.8 to 92.2)	7.3 (4.3 to 12.1)	1.0 (0.2 to 4.1)	3.4 (1.5 to 7.2)	187, 207
P value					p=0.018
Ethnicity					
White	84.5 (80.9 to 87.5)	9.0 (6.7 to 11.9)	2.8 (1.7 to 4.8)	3.7 (2.3 to 5.8)	481, 590
Black or Black African or Black Caribbean or Black British	60.7 (28.9 to 85.4)	29.6 (9.2 to 63.7)	0	9.7 (1.2 to 49.3)	11, 9
Asian or Asian British	68.3 (40.1 to 87.4)	15.1 (3.8 to 44.9)	9.5 (1.6 to 40.2)	7.1 (0.9 to 38.9)	15, 16
Mixed or multiple or other ethnic groups	56.6 (23.8 to 84.5)	27.7 (7.3 to 65.2)	15.6 (2.6 to 56.6)	0	10, 14
P value					p=0.025
Self-described sexual identity					
Heterosexual or Straight	83.3 (79.8 to 86.4)	9.8 (7.5 to 12.7)	3.1 (1.9 to 5.1)	3.8 (2.4 to 5.9)	493, 550
Lesbian, Gay, Bisexual or Other	66.5 (43.0 to 84.0)	21.0 (7.9 to 45.0)	6.1 (1.0 to 30.6)	6.3 (1.0 to 30.7)	21, 75
P value					p=0.017
Social grade					
A/B - Higher/intermediate managerial, administrative and professional	77.9 (69.5 to 84.5)	13.3 (8.3 to 20.7)	5.5 (2.6 to 11.3)	3.3 (1.2 to 8.6)	121, 158
C1/C2 - Supervisory, clerical and junior managerial, administrative and professional, skilled manual workers	84.3 (79.8 to 88.0)	8.9 (6.2 to 12.7)	2.1 (1.0 to 4.5)	4.7 (2.8 to 7.7)	306, 348
D/E - Semi-skilled and unskilled manual, casual, lowest grade and unemployed	84.0 (75.0 to 90.2)	10.5 (5.7 to 18.7)	3.8 (1.4 to 10.4)	1.6 (0.3 to 7.7)	94, 125
P value					p=0.19
Education level					
Degree	84.0 (79.2 to 87.9)	9.0 (6.1 to 13.1)	3.2 (1.6 to 6.1)	3.8 (2.1 to 6.9)	272, 331
Below degree	81.7 (76.3 to 86.1)	11.1 (7.7 to 15.8)	3.4 (1.7 to 6.6)	3.8 (2.0 to 7.1)	238, 288
No qualifications	73.6 (36.5 to 93.1)	21.9 (5.0 to 60.1)	0	4.5 (0.2 to 57.5)	10, 12
P value					p=0.79
Living together - Relationship but not living together - Single					
Married/steady and living together	84.6 (80.4 to 88.0)	9.4 (6.8 to 13.0)	1.8 (0.8 to 3.9)	4.2 (2.5 to 6.8)	352, 421
Married/steady not living together	78.3 (67.2 to 86.4)	13.1 (7.0 to 23.1)	6.1 (2.4 to 14.7)	2.5 (0.6 to 10.3)	73, 92
Casual, new, >1, at end or other*	84.0 (66.8 to 93.2)	7.7 (2.2 to 23.7)	8.3 (2.5 to 24.4)	0	34, 43
Single	77.1 (64.8 to 86.0)	12.6 (6.3 to 23.6)	4.8 (1.5 to 14.2)	5.4 (1.8 to 14.9)	62, 75
P value					p=0.16
Been furloughed under the Coronavirus Job Retention Scheme					
No	82.0 (78.0 to 85.4)	11.4 (8.7 to 14.8)	3.5 (2.1 to 5.8)	3.1 (1.8 to 5.3)	420, 511
Yes	85.7 (77.1 to 91.4)	5.6 (2.4 to 12.5)	1.7 (0.3 to 7.6)	7.0 (3.3 to 14.3)	97, 117
P value					p=0.042
Became unemployed					
No	83.5 (79.8 to 86.5)	9.7 (7.4 to 12.7)	3.2 (2.0 to 5.3)	3.6 (2.2 to 5.7)	476, 573
Yes	73.8 (57.9 to 85.3)	16.9 (8.0 to 32.1)	2.3 (0.3 to 16.3)	6.9 (2.1 to 20.6)	41, 55
P value					p=0.21
Days drinking in the last week (n)					
0	80.9 (75.0 to 85.7)	10.0 (6.6 to 14.9)	3.7 (1.8 to 7.3)	5.4 (3.0 to 9.4)	212, 250
1–2	83.4 (77.8 to 87.8)	11.3 (7.7 to 16.3)	2.4 (1.0 to 5.6)	2.8 (1.3 to 6.2)	216, 264
3–4	84.5 (72.9 to 91.7)	9.6 (4.3 to 20.2)	4.3 (1.3 to 13.7)	1.5 (0.2 to 11.1)	61, 74
5–7	86.3 (68.0 to 95.0)	5.8 (1.2 to 23.4)	3.2 (0.4 to 21.8)	4.6 (0.8 to 22.4)	30, 41
P value					p=0.78
Drinking habits compared with pre-COVID-19 outbreak					
Less these days	81.2 (74.1 to 86.6)	11.7 (7.4 to 17.9)	4.7 (2.3 to 9.5)	2.4 (0.9 to 6.6)	153, 195
About the same	83.7 (78.9 to 87.7)	9.0 (6.1 to 13.1)	2.2 (1.0 to 4.9)	5.0 (2.9 to 8.3)	272, 326
More these days	82.4 (73.2 to 88.9)	11.4 (6.3 to 19.7)	3.5 (1.2 to 10.0)	2.7 (0.8 to 8.9)	94, 109
P value					p=0.47
Current smoker					
No	83.5 (79.6 to 86.8)	9.9 (7.4 to 13.2)	2.8 (1.6 to 4.9)	3.7 (2.3 to 6.1)	410, 500
Yes	79.7 (71.0 to 86.3)	11.4 (6.6 to 18.9)	4.8 (2.0 to 10.8)	4.2 (1.7 to 10.1)	110, 130
P value					p=0.65
Symptoms of depression (PHQ-2 score)					
No symptoms of depression (0–2)	86.0 (82.0 to 89.3)	7.6 (5.2 to 10.9)	2.4 (1.2 to 4.6)	4.0 (2.4 to 6.7)	348, 421
Symptoms of depression (3–6)	76.5 (69.4 to 82.3)	14.9 (10.2 to 21.2)	5.1 (2.6 to 9.7)	3.6 (1.6 to 7.8)	166, 203
P value					p=0.012
Symptoms of anxiety (GAD-2 score)					
No symptoms of anxiety (0–2)	83.0 (78.5 to 86.7)	10.2 (7.4 to 14.0)	2.8 (1.5 to 5.3)	3.9 (2.3 to 6.7)	326, 387
Symptoms of anxiety (3–6)	82.5 (76.4 to 87.3)	10.3 (6.7 to 15.5)	3.5 (1.7 to 7.4)	3.7 (1.7 to 7.5)	191, 241
P value					p=0.96

Some 631 participants reported only or usually using a ‘more effective’ method of contraception in the year before the first lockdown. Users of emergency contraception only were classed as ‘stopped using contraceptives’. P values were calculated from F values generated from Pearson χ^2^ statistics using the second-order correction of Rao and Scott.^45^

*In a ‘casual’ relationship, in a ‘new’ relationship, in more than one relationship, recently ended a relationship or ‘other’ relationship status.

CI, confidence interval; GAD-2, Generalised Anxiety Disorder-2; PHQ-2, Patient Health Questionnaire-2.

Unmet needs for contraceptive services varied by sexual identity and markers of physical and mental health. Among all participants (n=1488), 29.3% (26.9%–31.8%) reported trying to access a contraceptive service between March 2020 and April 2021; 74 (16.4% (13.0%–20.4%) of those who tried to access) reported being unable to do so at least once (unmet need). Many of those also reported at least one successful access attempt; only 24 were unable to access a contraceptive service at all (online supplemental figure S2a; online supplemental table S3). Young participants were most likely to report an unmet need (7.4% (6.1%–9.0%) compared with those aged 35–44 years: 2.9% (2.1%–3.9%); figure 1), as were those from minoritised sexual identities (11.7% (10.0%–13.6%) compared with heterosexuals: 4.5% (3.5%–5.8%)). After adjustment for age, anxiety (adjusted odds ratio (aOR): 1.98 (1.16–3.36)) and depression (aOR: 1.67 (0.97–2.85)) were both associated with unmet need. Current smokers were also at higher risk of unmet need (aOR: 2.91 (1.71–4.96) vs non-smokers). Of barriers cited by those with unmet need (n=74), most related to clinic closures and appointment cancellations or unavailability (70.2%; online supplemental figure S2b).

Among all participants (n=1488), 13.6% reported a current pregnancy or pregnancy in the past year (n=199). The mean LMUP score for these pregnancies was 9.2 (standard deviation (SD): 3.0; with scores >9 classed as ‘planned’), and 6.6% (3.9%–11.1%) were scored as an unplanned pregnancy (LMUP score 0–3), while 33.3% of pregnancies (27.1%–40.2%) scored as ‘ambivalent’ (LMUP score 4–9) and 60.1% (53.1%–66.7%) scored as ‘planned’ (LMUP score >9). By comparison, among the 285 participants who reported a pregnancy between 1 and 5 years ago (but no pregnancy in the past year), the mean LMUP score was 8.6 (SD: 3.6; difference in weighted mean=0.58, p=0.064; data not shown), and 12.3% (8.8%–16.8%) were scored as unplanned, 30.7% (27.1%–40.2%) as ‘ambivalent’ and 57.0% (51.2%–62.7%) as ‘planned’. Eleven participants (6.1% of those reporting a pregnancy) reported an abortion in the past year, and six of these had a pregnancy that was scored as unplanned, though none of these participants reported unsuccessful attempts to access contraceptive services. Pregnancies in older participants were more likely than those in younger participants to be planned (difference in mean score for those aged 25–29 years: 2.90 (1.40–4.41), compared with participants aged 18–24 years; table 3). Cohabitation, relationship status and social grade were associated with pregnancy and pregnancy planning scores. Pregnancies were less commonly reported by those in non-cohabiting relationships compared with those living with partners (7.3% (4.1%–12.7%) vs 17.1% (14.8%–19.7%)). Single participants were less likely to report being pregnant (3.0% (1.1%–8.0%)); those who did were more likely to have an unplanned pregnancy compared with those in a non-cohabiting relationship (age-adjusted difference compared with single participants: 4.46 (2.39–6.53)) or a cohabiting relationship (age-adjusted difference: 5.31 (3.59–7.02)). Those working in less-skilled occupations, receiving state benefit or who were unemployed had lower LMUP scores. Smoking was associated with lower LMUP scores (age-adjusted score difference: –‍1.10 (–‍2.16 to –‍0.05)).

**Table 3:** Pregnancies in the past year and their ‘plannedness’ scored using the London Measure of Unplanned Pregnancy among sexually active participants aged 18–44 years

Parameter	Pregnancy in past year (% (CI))	Of which unplanned (% (CI)	Mean LMUP score (SD)	Age-adjusted difference in mean LMUP score (CI)	Denominator (weighted, unweighted)
Total	13.6 (11.9 to 15.6)	6.6 (3.9 to 11.1)	9.2 (3.0)	–	1280, 1488
Age (years)					
18–24	11.8 (8.1 to 16.9)	22.8 (10.5 to 42.7)	6.6 (3.3)	–	213, 265
25–29	16.3 (12.6 to 20.7)	6.0 (2.1 to 15.8)	9.5 (2.9)	2.90 (1.40 to 4.41)	324, 397
30–34	16.6 (12.6 to 21.5)	0	10.3 (1.9)	3.67 (2.27 to 5.07)	272, 320
35–44	10.9 (8.4 to 14.0)	5.1 (1.6 to 15.1)	9.3 (3.1)	2.70 (1.16 to 4.23)	470, 506
				p<0.0001	
Ethnicity					
White	12.2 (10.4 to 14.3)	6.6 (3.7 to 11.6)	9.3 (3.1)	–	1098, 1316
Black or Black African or Black Caribbean or Black British	24.5 (13.9 to 39.4)	13.6 (1.4 to 62.9)	8.3 (3.0)	0.21 (–1.82 to 2.25)	45, 35
Asian or Asian British	23.4 (15.3 to 34.1)	0	9.6 (1.9)	0.09 (–0.99 to 1.17)	79, 70
Mixed or multiple or other ethnic groups	17.5 (8.7 to 32.0)	15.0 (1.8 to 63.4)	8.1 (3.7)	−1.34 (–3.22 to 0.54)	44, 57
				p=0.55	
Self-described sexual identity					
Heterosexual or Straight	13.9 (12.1 to 16.0)	6.6 (3.8 to 11.2)	9.3 (3.0)	–	1224, 1325
Lesbian, Gay, Bisexual or Other	6.7 (2.0 to 20.1)	9.7 (1.1 to 50.3)	6.2 (3.0)	−2.51 (–3.99 to –1.02)	42, 151
				p=0.0011	
Social grade					
A/B - Higher/intermediate managerial, administrative and professional	16.4 (12.6 to 21.1)	2.8 (0.6 to 12.1)	10.1 (2.4)	–	291, 370
C1/C2 - Supervisory, clerical and junior managerial, administrative and professional, skilled manual workers	12.0 (9.7 to 14.6)	5.1 (2.0 to 12.4)	9.2 (3.0)	−0.81 (–1.69 to 0.08)	669, 730
D/E - Semi-skilled and unskilled manual, casual, lowest grade and unemployed	14.6 (11.1 to 18.9)	13.1 (6.2 to 25.6)	8.2 (3.3)	−1.36 (–2.49 to –0.22)	320, 388
				p=0.043	
Education level					
Degree	14.3 (11.9 to 17.2)	3.0 (1.0 to 8.7)	9.7 (2.6)	–	668, 771
Below degree	12.7 (10.2 to 15.7)	10.8 (5.6 to 19.8)	8.6 (3.4)	−0.73 (–1.61 to 0.15)	563, 661
No qualifications	14.9 (7.2 to 28.3)	12.3 (1.2 to 62.7)	8.9 (3.2)	−0.57 (–3.14 to 2.01)	48, 56
				p=0.25	
Living together - Relationship but not living together - Single					
Married/steady and living together	17.1 (14.8 to 19.7)	3.9 (1.8 to 8.0)	9.5 (2.8)	–	903, 1035
Married/steady not living together	7.3 (4.1 to 12.7)	10.0 (1.4 to 47.5)	8.0 (3.2)	−0.84 (–2.53 to 0.84)	155, 193
Casual, new, >1, at end or other*	5.4 (2.3 to 12.4)	31.9 (7.6 to 73.0)	6.9 (3.9)	−1.81 (–5.24 to 1.63)	94, 108
Single	3.0 (1.1 to 8.0)	74.6 (10.8 to 98.6)	2.7 (1.3)	−5.31 (–7.02 to –3.59)	127, 151
				p<0.0001	
Been furloughed under the Coronavirus Job Retention Scheme					
No	13.9 (12.0 to 16.1)	6.7 (3.7 to 11.7)	9.2 (3.0)	–	1055, 1225
Yes	12.2 (8.4 to 17.3)	6.8 (1.7 to 23.8)	9.4 (3.1)	0.76 (–0.36 to 1.88)	213, 251
				p=0.18	
Became unemployed					
No	13.7 (11.9 to 15.9)	6.5 (3.7 to 11.2)	9.3 (3.0)	–	1156, 1338
Yes	12.5 (7.5 to 20.0)	8.8 (1.5 to 38.5)	7.8 (3.0)	−1.37 (–2.98 to 0.25)	113, 138
				p=0.097	
Days drinking in the last week (n)					
0	20.5 (17.3 to 24.1)	5.3 (2.5 to 10.9)	9.4 (3.0)	–	550, 635
1–2	8.3 (6.2 to 11.1)	9.8 (3.8 to 22.7)	8.8 (3.3)	−0.51 (–1.58 to 0.55)	493, 575
3–4	9.2 (5.6 to 14.8)	10.5 (2.2 to 38.3)	9.0 (3.0)	−0.31 (–1.58 to 0.97)	158, 182
5–7	8.0 (3.6 to 16.7)	0	9.3 (2.8)	−0.35 (–2.40 to 1.71)	77, 94
				p=0.79	
Drinking habits compared with pre-COVID-19 outbreak					
Less these days	16.4 (13.0 to 20.6)	8.6 (4.0 to 17.3)	9.5 (3.2)	–	373, 449
About the same	13.5 (11.1 to 16.3)	4.6 (1.8 to 11.2)	9.2 (2.9)	−0.13 (–1.04 to 0.77)	667, 765
More these days	8.1 (5.2 to 12.6)	12.0 (3.0 to 37.4)	7.6 (3.2)	−1.34 (–2.85 to 0.17)	221, 255
				p=0.20	
Current smoker					
No	14.3 (12.3 to 16.6)	5.7 (3.0 to 10.5)	9.4 (3.0)	–	1002, 1162
Yes	10.7 (7.6 to 15.0)	11.5 (4.2 to 27.5)	8.0 (3.1)	−1.10 (–2.16 to –0.04)	275, 324
				p=0.042	
Symptoms of depression (PHQ-2 score)					
No symptoms of depression (0–2)	15.1 (12.8 to 17.7)	5.3 (2.6 to 10.5)	9.5 (2.8)	–	832, 955
Symptoms of depression (3-6)	10.8 (8.2 to 14.1)	10.4 (4.5 to 22.3)	8.3 (3.4)	−0.90 (–1.88 to 0.07)	434, 518
				p=0.070	
Symptoms of anxiety (GAD-2 score)					
No symptoms of anxiety (0–2)	15.2 (12.8 to 17.8)	5.3 (2.6 to 10.8)	9.5 (2.9)	–	783, 887
Symptoms of anxiety (3–6)	11.4 (8.9 to 14.6)	9.3 (4.3 to 19.2)	8.6 (3.3)	−0.70 (–1.60 to 0.19)	488, 593
				p=0.12	
Accessing contraceptive services and outcomes					
Did not try to access contraceptive services	14.5 (12.4 to 17.0)	5.9 (3.1 to 11.0)	9.4 (3.0)	–	916, 1047
Accessed services successfully	11.0 (7.9 to 15.0)	11.0 (4.2 to 26.1)	8.3 (3.3)	−0.69 (–1.72 to 0.34)	304, 367
Faced difficulty accessing services but was able to access in the end	15.6 (7.0 to 31.1)	0	10.2 (1.5)	1.11 (–0.33 to 2.54)	39, 50
Unable to access contraceptive services	9.5 (2.2 to 33.3)	0	10.5 (0.7)	0.11 (–0.79 to 1.00)	21, 24
				p=0.18	
Needed condoms but could not get hold of them					
No	13.2 (11.4 to 15.2)	6.0 (3.3 to 10.6)	9.4 (3.0)	–	1188, 1383
Yes	20.8 (13.1 to 31.2)	13.0 (3.3 to 39.4)	8.0 (2.9)	−0.88 (–2.14 to 0.38)	79, 92
				p=0.17	

The London Measure of Unplanned Pregnancy (LMUP) score scores each pregnancy on a range of 0–12 to represent the relative plannedness of the pregnancy. Differences for LMUP score across all categories other than age are adjusted for age using a linear regression model.

*In a ‘casual’ relationship, in a ‘new’ relationship, in more than one relationship, recently ended a relationship or ‘other’ relationship status.

CI, confidence interval; GAD-2, Generalised Anxiety Disorder-2; LMUP, London Measure of Unplanned Pregnancy; PHQ-2, Patient Health Questionnaire-2; SD, standard deviation.

## Discussion

Our study used a large quasi-representative sample of the British population and emphasises the high level of need for contraceptive services that continued during the year following the first national COVID-19 lockdown. One in six participants reported an unmet need in attempting to access contraceptive services, with clinic closures, suspension of face-to-face appointments and disruptions to travel reported as the reasons. However, most participants were successful in accessing services and those reporting difficulties also often reported successful attempts, suggesting that resilience and adaptability in service delivery mitigated some of the challenges. Overall, 53.9% of participants reported using an effective method as their usual contraception during the pandemic, and this proportion was similar to the 56.4% and 54.2% using effective methods found in the previous Natsal-2 (2000–2001) and Natsal-3 (2010–2012) surveys, respectively.^31^ However, we found that low proportions of participants from Asian or Asian British ethnic backgrounds (25.9%) or from a mixed or multiple or other ethnic background (27.5%), compared with participants from White ethnic backgrounds (58.1%), reported using effective methods. Though likely due at least partly to pre-pandemic differences,^32^ this suggests different levels of risk for unplanned pregnancy by ethnicity during this national period of high stress and uncertainty. It was reassuring that most participants (82.8%) using effective contraception pre-pandemic reported not having to switch method or stop using contraception because of the pandemic, and 10.2% reported switching but were able to use similarly or more effective methods. However, consistent with earlier research, we found that younger participants, while being at greater risk of unplanned pregnancy, were more likely to have switched method because of the pandemic, and to report barriers to accessing contraceptive services.^16^ Routinely collected data indicate large reductions in contraception prescription and dispensing in England in 2020 compared with 2019.^33–35^ Our data suggest that difficulties accessing services, primarily due to closures and appointment cancellations, may have contributed to this reduction.^9^

Our analysis of pregnancies during the pandemic builds on and challenges previous research. Elsewhere we report that compared with Natsal-3 data collected a decade previously (in 2010–2012), pregnancies and abortions in the first year of the pandemic were substantially lower.^18^ We also found that pregnancies during the pandemic were less likely to be scored as unplanned compared with a decade previously (6.2% vs 18.3%).^18^ These observations correspond with several proposed mechanisms.^36^ On the one hand, ongoing improvements in service provision may have impacted on access to contraceptive methods, especially LARCs. On the other hand, less sexual contact during the pandemic might have led to lower pregnancy rates,^37 38^ reducing risk of unplanned pregnancy regardless of contraception and service access. Our finding that EC use was lower during the pandemic than in preceding year, with a small but not significant increase in plannedness score of pregnancies, is consistent with the hypothesised effects of reduced social contact on sexual behaviour leading to unplanned pregnancy. Other studies report both desires to postpone pregnancy, most commonly citing fear or uncertainty over service access, and bringing forward pregnancy plans due to changed circumstances,^39–41^ mechanisms potentially contributing to an overall increase in plannedness.

In contrast to our findings indicating a decrease in unplanned pregnancies during the pandemic, a convenience sample cohort study of pregnant women in the UK found that conceptions in the year following the first lockdown were more likely to be unplanned than pre-lockdown conceptions.^14^ Two study design factors might explain the different results. The cohort study used online adverts to recruit participants, which might introduce bias. Additionally, it only included participants who were still pregnant at the time of recruitment (commencing May 2020), thus excluding unplanned pregnancy terminations before the end of April 2020. The Natsal-COVID estimate, recruiting a wider range of participants and using weighting to achieve representativeness, seems less susceptible to bias.

In our quasi-representative population sample, several markers of vulnerability and health risk behaviours were associated with elevated reproductive health risks. While patterns of risks during the pandemic may match existing inequalities, pandemic-induced inequalities in access to contraceptive services may have exacerbated these. Participants with poor health and behavioural risk factors such as smoking and drinking alcohol reported higher rates of unmet need for contraceptive services and higher rates of switching or stopping contraceptives. Participants in lower social grades and who smoked were more likely to report unplanned pregnancies, which is similar to patterns previously observed in the UK.^7 42^ While these findings suggest a greater risk of unplanned pregnancy in these groups, we were unable to directly link the ‘plannedness’ of each pregnancy to specific attempts to access contraceptive services. In our study, most people switching contraceptive method switched to a similarly or more effective method, suggesting flexibility and adaptability in participants’ responses to changing service provision, which might have been sufficient to meet contraceptive needs in many cases. Our findings are also consistent with convenience-sample evidence from the USA that a drop in the desire to be pregnant was associated with low income, but not independently associated with decreased income due to the pandemic.^40^ Natsal-COVID benefited from a questionnaire design and approach developed by the team responsible for the decennial Natsal survey to obtain rigorous data on potentially sensitive behaviours and experiences.^19^ Natsal-COVID included a large, national sample and used quota-based sampling and weighting to improve representativeness. Unlike the decennial Natsal survey, Natsal-COVID was not a probability sample, and is therefore not directly representative of the general population.^43 44^

Our study informs adaptations to contraceptive services to meet patient needs and preferences, including in the pandemic recovery phase. Regardless of differences in how health systems are structured, financed or commissioned in other high-income countries, our findings broadly indicate the likely impacts of the pandemic on contraceptive method and service use. We highlight here inequalities across age, ethnicity, social disadvantage and mental health. Ongoing provision of contraceptive services and future crisis planning should ensure ease and equality of access to contraceptive services for all to address the impact on contraceptive method choice and availability.

## Data Availability

Data are available in a public, open access repository. https://beta.ukdataservice.ac.uk/datacatalogue/studies/study?id=8865.
